# Evaluating a multicomponent social behaviour change communication strategy to reduce intimate partner violence among married couples: study protocol for a cluster randomized trial in Nepal

**DOI:** 10.1186/s12889-016-3909-9

**Published:** 2017-01-13

**Authors:** Cari Jo Clark, Rachael A. Spencer, Binita Shrestha, Gemma Ferguson, J. Michael Oakes, Jhumka Gupta

**Affiliations:** 1 Hubert Department of Global Health, Rollins School of Public Health, Emory University, 1518 Clifton Road NE, Office 7033, Atlanta, GA 30322 USA; 2 Department of Behavioral Sciences and Health Education, Rollins School of Public Health, Emory University, Atlanta, GA USA; 3 Equal Access International, San Francisco, CA USA; 4 Department of Epidemiology and Community Health, School of Public Health, University of Minnesota, Minneapolis, MN USA; 5 Department of Global and Community Health, College of Health and Human Services, George Mason University, Fairfax, VA USA

## Abstract

**Background:**

Intimate partner violence (IPV) is a significant public health issue that affects 1 in 3 women globally and a similarly large number of women in Nepal. Over the past decade, important policy and programmatic steps have been taken to address violence against women in Nepal. There remains a dearth of evidence on the effectiveness of primary violence prevention strategies. The Change Starts at Home study begins to fill this gap by utilizing a multi-component social behaviour change communication (SBCC) strategy involving a radio drama and community mobilization to shift attitudes, norms and behaviours that underpin IPV perpetration in Nepal.

**Methods/Design:**

The study uses a concurrent mixed-methods design. The quantitative aspect of the evaluation is a pair-matched, repeated cross-sectional 2-armed, single-blinded cluster trial (RCT: *N* = 36 clusters, 1440 individuals), comparing a social behaviour change communication (SBCC) strategy to radio programming alone for its impact on physical and / or sexual IPV at the end of programming (12 months’ post-baseline) and 6-months post the cessation of project activities (18-months post baseline). The qualitative aspects of the design include several longitudinal approaches to understand the impact of the intervention and to examine mechanisms of change including in-depth interviews with participants (*N* = 18 couples), and focus group discussions with community leaders (*N* = 3 groups), and family members of participants (*N* = 12 groups). Treatment effects will be estimated with generalized logistic mixed models specified to compare differences in primary outcome from baseline to 12-month follow-up, and baseline to 18-months follow-up in accordance with intention-to-treat principles.

**Discussion:**

The study rigorously evaluates the effectiveness of a promising strategy to prevent IPV. The results of the trial will be immediately useful for governmental, nongovernmental, and donor funded programs targeting partner violence or social norms that underpin it. Findings of the study will also contribute to global knowledge on the effectiveness of media and community engagement as a primary prevention strategy for IPV.

**Trial registration:**

Trial was registered in clinicaltrials.gov, NCT02942433, 10/13/2016, retrospectively registered.

## Background

Intimate partner violence (IPV) is a significant public health issue that affects 1 in 3 women globally [1] and a similarly large number of women in Nepal [2, 3]. Over the past decade, important policy and programmatic steps have been taken to address violence against women in Nepal [4, 5]. The 2006 Gender Equality Act amended 56 discriminatory provisions in the law and clarified and expanded definitions of violent crimes committed against women, including rape and homicide. In 2009, the government of Nepal passed the Domestic Violence (Crime and Punishment) Act, which renders illegal violence committed by one family member against another. Resources aimed at supporting women who experience violence and preventing violence against women and girls (VAWG) have also increased, and both government and civil society organizations have opened centres throughout the country [5]. There remains, however, a dearth of evidence on the effectiveness of primary violence prevention strategies (i.e. strategies that prevent violence before it occurs) and existing evidence is skewed toward secondary and tertiary prevention strategies (i.e. strategies that aim to respond to violence after it has taken place and/or mitigate its consequences) developed in high-income countries [6]. Equal Access’ (EA) Change Starts at Home Project (*Change*) begins to fill this gap by utilizing a multi-component social behaviour change communication (SBCC) strategy involving a radio drama and community mobilization to shift attitudes, norms and behaviours that underpin IPV perpetration in Nepal.

Existing literature suggests that interventions designed to change social norms can positively influence individual attitudes and practices around IPV [7]. Multicomponent interventions incorporating radio programs have shown increased knowledge and awareness about IPV, [8, 9] decreased endorsement of gender-inequitable attitudes, [8–10] and increased joint household decision making [9] and communication about partner violence [8] and sex [9, 10]. While no prior studies have investigated the role of radio on the prevention or reduction of IPV in Nepal, radio dramas have been shown to have effects on ideation and behaviour change associated with other gendered health behaviours, such as family planning utilization [11].

Despite the small but growing body of literature on the utility of media-based social norms interventions in preventing VAWG, there have been few rigorous studies of violence prevention interventions conducted in Nepal. From August 2007 to May 2010, EA implemented the *Voices* project, which addressed the twin pandemics of HIV (human immmunodeficiency virus) and VAWG using radio and community outreach. The evaluation of *Voices*, funded by the United Nations Trust Fund to End Violence Against Women, demonstrated that the project successfully increased dialogue between husbands and wives around sexual relations and HIV, understanding of legal issues related to VAWG, understanding that marital rape is illegal, intervention in cases of IPV, support for actual help-seeking behaviours, and decreased women’s tolerance of VAWG [12]. However, the prior evaluation did not utilize a control group, did not involve members of intact couples, and did not directly measure changes in norms but instead measured individual attitudes.

The current iteration of this approach to violence prevention programming and evaluation address these gaps by building on existing social norms change literature and experimentally testing a promising, multi-component communication-based, social behaviour change intervention (*Change*). *Change* seeks to address social norms, especially the acceptability of IPV, which plays a powerful role in shaping individuals’ attitudes and behaviours, including the perpetration of IPV and responses to victims of violence [7, 13]. This intervention posits that changes in social norms can successfully promote sustainable change that protects women from IPV [14]. Specifically, the trial seeks to: a) assess whether the multicomponent *Change* program (i.e., media + community engagement strategy) yields a greater reduction in cluster-level IPV rates compared to the *Change* radio program alone; b) determine whether any potential reductions in cluster-level IPV rates are sustained 6 months after cessation of intervention activities, c) explore what mechanisms and factors may explain any differences that may or may not be detected; and d) identify potential socio-demographic or contextual factors that may moderate the impact of the *Change* program. Secondarily, the trial also expects to observe improved conflict resolution techniques, couple communication, attitudes toward gender equity and acceptability of IPV, and perceptions of community acceptance of IPV among *Change* intervention communities versus communities receiving the *Change* radio programming alone. As the target of the intervention are social norms underpinning individual behaviour, a cluster design is utilized.

## Methods/design

**Fig. 1:**
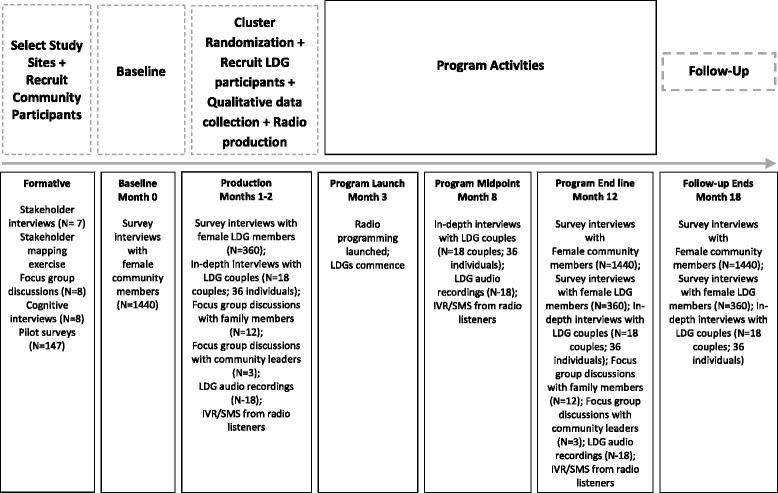
Research Design

The study utilizes a concurrent mixed methods design which capitalizes on the strengths of both qualitative and quantitative data collection techniques to ascertain project effectiveness. This study design will also document participant and stakeholder experiences, as well as investigate the mechanisms of action and contextual factors that could affect the outcomes and/or explain results [15]. The quantitative aspect of the evaluation is a pair-matched, repeated cross-sectional 2-armed, single-blinded trial (RCT: *N* = 36 clusters [Village Development Committees)], 1440 individuals), comparing a social behaviour change communication (SBCC) strategy to radio programming alone for its impact on physical and / or sexual IPV at the end of programming (12 months post-baseline; February-March 2017) and 6-months post the cessation of project activities (18-months post baseline; September-October 2017). The qualitative aspects of the design include several longitudinal approaches to understand the impact of the intervention and examine mechanisms of change, including in-depth interviews with participants (*N* = 18 couples), and focus group discussions with community leaders (*N* = 3), and family members of participants (*N* = 12). Measurement activities are timed (Fig. 1) to enable iterative and parallel data analysis. The combined methodology, along with data collected through ongoing monitoring of intervention activities, will offer a rigorous test of the intervention and provide information to inform scale-up and replication efforts.

### Population

**Fig. 2:**
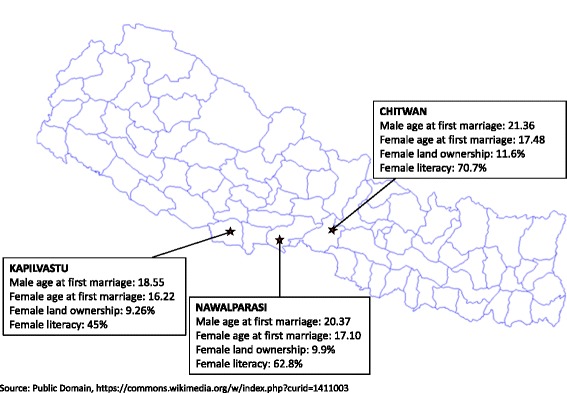
Study Districts

The study is set in the three districts of Nepal including Nawalparasi, Kapilvastu, and Chitwan. All three districts are over 80% Hindu and have similar profiles in terms of ages at first marriage and levels of female land ownership, but levels of female literacy are more variable (Fig. 2) [16, 17]. These districts were selected because they are located in the region (*terai*) with the highest IPV prevalence,^2^ and these districts are areas in which the local implementing partner, *Vijaya Development Resource Centre* (VDRC), has extensive contacts on the ground and a strong local reputation to facilitate a welcoming and safe atmosphere for the proposed research.

#### Eligibility criteria

Eligible VDCs (unit of randomization) include those that are at least 30 – 40 kilometres in distance from one another, have separate major markets and major health centres, and are similar socially and economically (predominant caste, language, level of conservatism) to at least one other VDC in the sample as determined by the implementing partner. VDCs in which the implementing partner has existing relationships or the ability to develop relationships with community stakeholders and gatekeepers will be prioritized for selection.

Female survey participants are eligible if: 1) they are of reproductive age (between 18-49 years), 2) their husband is at least 18 years of age, 3) both the wife and husband reside regularly in the study area, and 4) the wife and husband live together. In addition to the eligibility criteria outlined for the survey participants, participants in treatment group activities must also commit to 9 months of weekly programming. Family members are eligible to participate in the study if they are 1) at least 18 years of age, and 2) a family member of a participant in treatment group activities. Community leaders are eligible to participate in the study if they are 1) at least 18 years of age, and 2) considered to be in a position of authority or influence within the study communities per the recommendation of other community stakeholders. Facilitators for intervention activities must: 1) have a 12th grade standard academic qualification or higher, 2) be living with his/her spouse, 3) have good communication skills, 4) have a good reputation in the community based on feedback from local social mobilizers, and 5) be able to commit to 9 months of programming and weekly reporting. Across activities, participants are ineligible if they cannot communicate in Nepali, have plans to relocate in the coming 2 years, or have an easily detectable physical or cognitive impairment that would preclude their participation.

#### Interventions

*Change* is a multi-component SBCC strategy designed to shift attitudes, norms and behaviours that underpin the power imbalances that disfavour women and increase women’s vulnerability to husbands’ IPV within couples in Nepal. Recognizing the social ecology of change, the intervention engages actors across multiple domains of influence, such as family members and community leaders, in addition to the primary target audience of married reproductive age women and their husbands. As a SBCC strategy, the intervention approaches IPV prevention through three key approaches: advocacy, social mobilization and behaviour change communication [18]. The behaviour change communication component is a 9-month, weekly radio drama with listener engagement through interactive voice response (IVR) and short message service (SMS), to which both the intervention and control conditions are exposed. The intervention communities are further engaged in radio Listening and Discussion Groups (LDGs), through which the male and female participants meet to critically reflect on the content of the radio episode through a curriculum-based process of guided discussion, in-group and home-based activities. LDGs serve as venues for life skills building and act as a platform through which community outreach activities are planned and executed, alongside local leaders who receive training and support to act as advocates in the community for more equitable social norms. The use of multiple modalities is in keeping with current best practices in social norms change [13] which recognizes that modifying complex phenomena (such as social norms) requires a multi-faceted approach.

### Theoretical orientation and phases

**Fig. 3:**
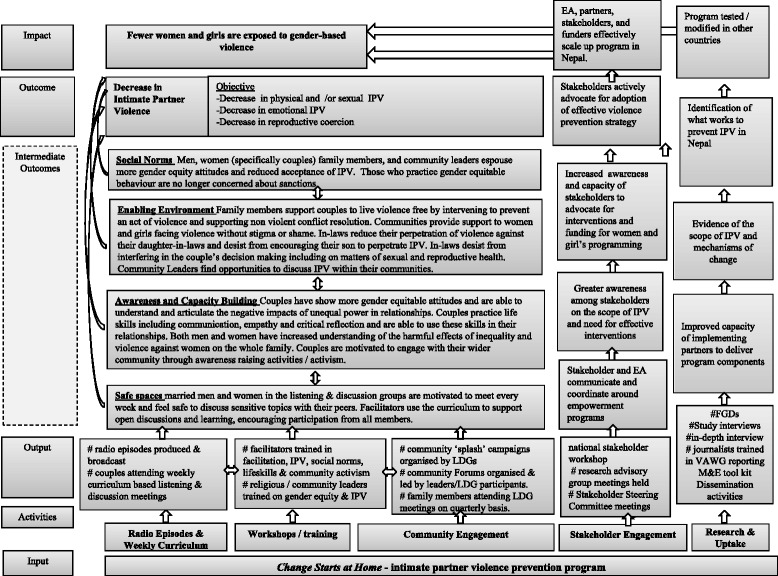
Change Starts at Home Theory of Change

**Fig. 4:**
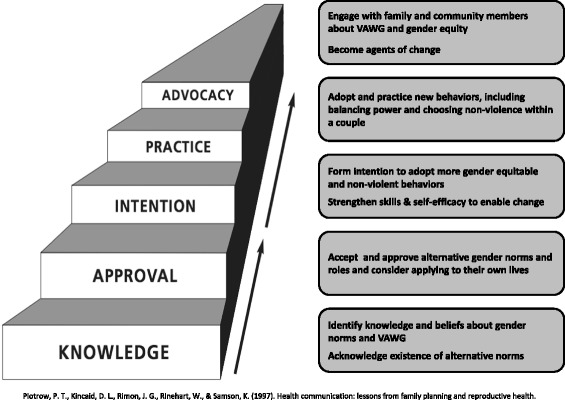
Attitude and Behaviour Change Process

The *Change* communication and mobilization intervention relies on a number of theoretical models including: the Socio-Ecological Model [18] to conceptualize the multiple contexts and factors that influence behaviour change, the Steps to Behaviour Change Framework, [19] and the Integrative Model of Behaviour Prediction [20] to anchor project activities and curriculum to particular stages and entry points toward behaviour change, and the Diffusion of Innovations theory [21] to guide efforts to extend the impact of the intervention beyond those most directly exposed (see Figs. 3 and 4 [19] for visual representations of the hypothesized change process). Collectively, the theoretical underpinning of the project recognizes that change is a process, although not necessarily linear, that occurs within embedded contexts of interpersonal, social and political contexts. The intervention is divided into 3 phases, each lasting 3 months and each delivered via the radio program and the curriculum-based weekly LDG sessions.

#### Phase 1: knowledge and approval

Current behavioural, normative and control beliefs related to gender norms and VAWG are identified and questioned through a mix of information, role modelling and discussions that highlight the diversity within ‘normative’ behaviour. During this phase, couples (both as listeners to the radio program and participants in the LDG sessions) are asked to not only acknowledge the existence of a range of behavioural, normative and control beliefs, but to also approve of and internalize those alternatives for themselves. By asking people to accept (and consider applying) new norms and roles, phase 1 will begin to shift negative attitudes related to acceptability of VAWG towards positive attitudes of acceptability of non-violence.

#### Phase 2: intention

The knowledge and skills gained in phase 1 enable couples to form a strong intention towards adopting more positive behaviours. They now have a more positive attitude towards a gender equitable and non-violent relationship and no longer subscribe to perceived negative social norms related to what is expected of them as a couple. The focus on life skills-based information and education (through both the radio program and LDGs) in this phase enables couples to not only accept that alternative norms and roles exist, but to develop beliefs and skills (example: good communication, empathy, critical thinking, self-awareness) necessary to begin achieving more equitable power in their own relationships. The life skills focus of the curriculum and radio programming therefore plays an important role to build self-efficacy, recognized as a key component in promoting sustained behaviour change. By this phase, those couples who are part of the LDGs should also begin to experience the positive benefits of having a new reference group / positive social identity supporting them to make good decisions as individuals and as a couple, which draws on Jackson’s assertion that social learning supports behaviour change [22].

#### Phase 3: practice and advocacy

Couples move through intention to actually changing behaviours. As per the integrative model, alongside intention and skills, behaviour change is most likely to occur if people have the necessary environment required to support the change. Therefore, whilst the primary beneficiaries of *Change* are couples, family and community members are also targeted (through community activities and the radio program) to ensure they provide an enabling environment and serve as catalysts for change. Community leaders will be trained to incorporate IPV prevention and response into their work as respected members of their communities and family members of LDG participants will be invited to special family-oriented LDG sessions alongside community-based events, such as community theatre, town hall meetings etc. Together, these activities at the various levels of society are hypothesized to affect beliefs, behaviours, and perceptions of social norms to reduce IPV (Table 1).

**Table 1:** Treatment and control conditions for change starts at home

Treatment	Control
Behaviour Change Communication	Behaviour Change Communication
o 9-month weekly episode behaviour change radio drama with IVR/SMS listener engagement	o 9-month weekly episode behaviour change radio drama with IVR/SMS listener engagement
Community Mobilization	
o Listening and Discussion Groups, 2 male and 2 female groups per study site meet weekly for 39 weeks (Village Development Council; VDC)	
o Training on gender equity, IPV, life skills, community mobilization, non-violent conflict resolution for LDG facilitators to support knowledge and skill set acquisition of LDG members	
o Joint couple’s session every month to foster mutual learning and understanding	
o Community mobilization incentives led by LDGs	
o Encouragement of family members of LGD members to listen to the radio and attend LDG sessions once every 3 months.	
o Awareness raising and street theatre to engage families and community members	
Advocacy	
o Training and 6-month follow up of religious/community leaders	
o Religious leaders support community mobilization activity of local LGD	

### Change components

#### Behaviour change communication

Individuals in study communities (both intervention and control) are reached through radio programming that is entertaining and educational – a form of “edu-tainment” – that models non-violent behaviour and supports acquisition of life skills. Media is an appropriate backbone to the intervention given its ability to “…inform, enable, motivate, and guide people to effect personal and social changes” [23]. It accomplishes these tasks by delivering information to large audiences, serving as a venue for voice, dialogue, and participation, and modelling desired health behaviours [24, 25]. Health behaviours can be impacted by media via both direct and indirect routes [23, 26]. Media can impact health behaviour directly by invoking an emotional or cognitive response, reducing perceived obstacles, revealing unhealthy norms, and creating a connection between valued emotions and desired behaviour change. Indirectly, media can bring about behaviour change by stimulating discussion between unexposed individuals and those who have been [26]. Change may also be entirely socially mediated, catalysing broader public discussion and policy change [23].

Radio is a widely accessible media form in countries throughout the world, including Nepal [2, 27, 28]. While no prior studies have investigated the role of radio on the prevention or reduction of IPV in Nepal, radio dramas have been shown to have effects on ideation and behaviour change associated with other gendered health behaviours, such as family planning utilization [11]. The name of the radio programming broadcast during *Change* is called *Samajhdari* which is Nepali for mutual understanding.

*Samajhdari* is a 30-min drama focused around a highway hotel along the East West Highway in Nepal. The hotel is run by a couple who, comparatively, have a positive and gender-equitable relationship. The wife handles financial issues in the hotel; whereas the husband takes care of the customers and works in the kitchen. Their hotel is popular among travellers passing by and the couple welcome different characters who meet to discuss and exchange stories about problems related to marriage, power and gender identities. Local voices in the form of voxpops,^1^ interviews, and case studies are weaved in to the drama as visitors to the hotel. After the drama, there is a summary segment which summarizes the overall discussion of the program and showcases the IVR responses sent to the program from listeners.

#### Community mobilization and advocacy

While promising, an important concern with utilizing radio programming alone to change behaviours is that radio consumption is largely passive, and thus may not necessarily yield behaviour change [29, 30]. Thus, in addition to radio programming, the project engages and mobilizes community members within radio-listening groups to promote discussion, reflection, and critical thinking around radio-broadcasted messages to facilitate behaviour change [31–33]. In the Change project, sex-separate LDGs will meet to dialogue about the radio programming and to create “homogenous, tightly knit groups in which there is private dissent against the current norm” [13] and support for the members’ integration of messages into everyday life [34]. LDGs will also receive a tool kit and financial resources to hold awareness-raising events in their communities. Direct engagement of community leaders, family members, and friends of LDG members assists in facilitating the integration of the new social norms [13]. While rigorous evaluations of the effectiveness of utilizing media in combination with dialogue groups and community mobilization to reduce IPV and change underlying gender norms are scarce, quasi-experimental research on other “gendered” health issues (e.g. family planning, HIV discussion) have been encouraging [35, 36].

#### LDGs

LDGs play a vital role in ensuring that the radio content remains relevant and applicable to the listening audience and provides a peer-to-peer group within which it is safe to explore with greater depth some of the issues raised in the radio program. EA trains facilitators that convene discussion groups following a weekly curriculum. Through facilitator feedback forms and monitoring visits, EA collects information and records listener reactions to the radio episodes, capturing perspectives on issues, as well as valuable feedback on which elements were of most interest to listeners, areas for improvement, questions, and requests for additional information. This feedback is then shared with EA’s production team to guide the content of future episodes.

Each session is guided by a step-by-step curriculum. Approximately 10 married couples meeting eligibility criteria will be recruited per ward (1 male and 1 female group per ward, for a total of 4 LDGs per VDC). Each group is managed by a trained facilitator of the same sex who convenes the group once weekly for approximately 2 h in a location deemed socially acceptable and physically accessible. At the start of Phase III each team will be provided with a tool kit to support community interactions, including a gender-focused film and discussion guide for community screenings, a video recording of the gender-focused street theatres (below) plus discussion guide, audio from select radio sessions plus discussion guides, and printed materials including a poster explaining the project. Each LDG will use the tool kit as appropriate for their community activities in Phase III.

#### Street theatre

The purpose of the street theatre is to allow large numbers of community members to be exposed to messaging that focuses on gender equity, and to encourage them to interact with the issues through the drama. A professional theatre company will be hired to script and direct a short, interactive, play. The play will be performed in one intervention site in each of the 12 districts and will also be professionally recorded so that other communities without access to live performances can view the screenings.

#### Community/religious leader workshop

Community leaders will be trained in a 2 day workshop designed to: 1) introduce the project 'Change Starts at Home’ to key stakeholders who are knowledgeable and supportive of the project 2) facilitate smooth implementation of project activities by getting community leader buy-in; 3) strengthen ties between EA/VDRC and the religious/community leaders to encourage better coordinated responses to violence against women, especially IPV in the targeted communities; and 4) provide a forum for the religious and community leaders to reflect on their own position and capacity to comprehend and respond to VAWG, especially IPV. During this workshop the leaders will generate an action plan designed to contribute to violence prevention and enhanced response to partner violence in their local communities; this action plan will be followed up by VDRC after 6 months. The religious community / religious leaders and their local LDG will also be encouraged to organize a joint event during phase III.

### Control group

The control group participants will be exposed to the radio program only, and will not participate in the LDGs, nor will they or their family members participate in the workshops and community activities. At three separate time points, a randomly selected sample of female community members who meet inclusion criteria will be invited to take a survey. To adhere to international standards of ethics surrounding conducting research with violence against women, female participants in the control group will be provided with the same referrals to free or low-cost resources as those in the intervention group [37].

### Intervention adherence

Intervention adherence is monitored through a number of mechanisms including:

1. The submission of weekly forms by the LDG facilitators, which includes attendance and an assessment of the conduct of the group;
2. Audio calls placed by Equal Access staff (at least 1 male and 1 female) to obtain more in-depth feedback on the conduct of the group;
3. Review of between 2 and 6 audio recordings of LDG sessions by Equal Access staff;
4. In-person support and monitoring visits by VDRC field officers to between 4 – 5 LDGs weekly; and
5. Booster trainings for LDG facilitators every 6 weeks.

### Concomitant interventions

The study team attempted to choose VDCs where other projects were not occurring or forthcoming prior to baseline. However, development aid to Nepal, especially following the earthquake in 2015, is not within the control of the current investigation. Study participants are not disallowed participation in other programs as withholding opportunities for material or other support would be potentially unethical and would not serve the underlying goals of *Change*. Therefore, the study team will monitor local, national, or international projects that might begin operating during the study period in all of the study communities. Further, a member of the National Health Research Council is among the study’s scientific advisory board, providing an opportunity to learn about other projects that are forthcoming, as all health-related projects require approval from the council to operate in the country.

### Outcomes

The primary and secondary outcomes, and covariates are assessed through an interview-administered survey at baseline, 12 months’ post-baseline and 18-months post baseline. Scale items and their sources are listed in Table 2.

**Table 2:** Outcomes and other measures for the change starts at home project

Outcomes	Source
Primary	
Physical and / or sexual intimate partner violence in prior 12 months	Standard Outcomes for Assessment of Intimate Partner Violence for What Works to Prevent Violence Global Program [49]
Secondary	
Psychological partner abuse in prior 12 months	World Health Organization Multi-Country Study (WHO MCS) on Women’s Health and Domestic Violence [50]
Economic partner abuse in the prior 12 months	United Nations Multi-country Study on Men and Violence [51]
Conflict and conflict resolution techniques in prior 12 months	Index of items from several studies. Item on quarrelling from WHO MCS [50]; two items adapted from the Relationship Self Efficacy Beliefs’ Scale [52]
Couple communication in prior 12 months	WHO MCS [50]
Attitudes toward gender equity	Adapted Gender Equitable Men (GEM) scale [53]
Attitudes toward the acceptability of intimate partner violence	Adapted GEM scale [53]
Perceptions of community-norms on the acceptability of partner violence	Developed for study
Other Measures	
Socio-demographics	Developed for the study
Help-seeking for IPV	Developed for the study
Exposure to violence from natal and marital family members	Developed for the study
Depressive symptoms	PHQ-8 [54]
Disability	Washington Group on Disability Statistics [55]
Exposure to messages on VAWG	Developed for the study
Participant reaction to the study	Reactions to Research Participation Scale [56]

### Quality control and data entry

Data quality procedures are outlined in the field manual in detail and include the selection of enumerators who have prior experience collecting data on sensitive topics, with special emphasis on VAWG, extensive classroom and field training and practice, pilot testing of the data collection application to limit data collection and entry errors, and direct observation, visual checks of the application entries, and in-person and telephone contact with a subset of entries to ascertain the veracity of the data collected. Daily field team debriefings and back-end data quality checks by the data collection firm and study team are used to identify and rectify errors in a timely manner. An audit trail will be kept of all changes made during data cleaning. After the first round of cleaning is complete, typically within one month of data collection, no further changes to the database will be allowed.

### Qualitative instruments

The overall goals of the qualitative assessments are to examine pathways through which outcomes of interest may be impacted by the *Change* Intervention and to assess contextual factors influencing intervention effectiveness. These will be achieved through in-depth interviews with participating couples and focus group discussions with LDG members’ family members and community leaders. The specific qualitative approach, research goal, audience, and time point have been summarized in Table 3.

**Table 3:** Schedule of qualitative measurements and objectives

Audience	Time Point	Qualitative Approach	Objectives
Couples in LDGs	Prior to the commencement of programming, midpoint, program end line, and 18 month follow-up	18 In-depth interviews each measurement occasion	To understand changes in knowledge, attitudes and behaviours, environmental facilitators and constraints to the couple exhibiting more gender equitable attitudes and behaviours, and the degree and impact of their activism within their community.
Family Members of LDG participants	Prior to the commencement of programming and program endline	12 Focus group discussions each measurement occasion	To understand changes in family-based norms on gender equity and intimate partner violence, and the impact of any project activities and couple-based activism they may have been exposed to.
Community Leaders	1st quarter of the project and 6 months thereafter	3 Focus group discussions each measurement occasion	To examine changes in their attitudes on gender equity and intimate partner violence, community norms on gender equity and intimate partner violence, contextual factors that are occurring in the community, and the impact of the project at the community level.
Radio Listeners	Quarterly	IVR/SMS listener feedback analysis	To examine change over time in community norms on gender equity and IPV.
LDG Members	1^st^ month of programming, midpoint, and program end line	6 LDG groups followed over 3 time points	To investigate change over time in attitudes and group norms on gender equity and IPV, and how the group process may facilitate these changes.

### Sample size and power

This study is powered to detect meaningful differences in prevalence of IPV. To calculate study power, we took into consideration the number of experimental conditions, the number groups/clusters nested within conditions, the number of persons within groups, the intra-class correlation coefficient (ICC), within-group over time correlations, and traditional variance components [38]. We have 36 units of randomization (VDCs) and 40 randomly sampled persons within each unit, 20 in each of two selected VDC sub-units called wards. Assuming a conservative over-time group-level correlation of 0.40, a 12-month prevalence of physical or sexual IPV of 14%, which was based on estimates from the 2011 Demographic and Health Survey in Nepal [2]. a conservative standard deviation of 25%, a conservative intra-class correlation of 0.05, with 80% power and 5% alpha (2 sided test) the study can detect a difference in percentage of 7%.

### Sampling frame and recruitment

EA, in consultation with VDRC, selected 12 VDCs per district (total of 36 clusters) where it was feasible to carry out project activities and minimize potential contamination. Within the VDC, two wards were randomly selected using probability proportionate to size methodology among eligible wards, defined as having a total household population between 100 and 550, a size assumed appropriate for project activities. Within each ward, a VDRC representative visited ward subdivisions, comprised of approximately 15-20 households to compile a list of households and identify those containing eligible couples based on information from key informants such as female village health workers and where available, existing lists. These household lists were aggregated at the ward level to create the project’s sampling frame.

### Community survey recruitment

For the survey, 40 women per VDC were randomly chosen from the list of households with eligible couples, 20 from each ward were then selected using simple randomization. For this process, within each ward, random numbers were generated in Excel. Households were subsequently organized from highest to lowest based on the randomly generated number. The 20 households with the highest randomly generated numbers were selected for the study. Ten possible replacements were similarly identified given the increased likelihood of ineligible couples based on the reliance on informants and other sources during the creation of the sampling frame.

### LDG participant recruitment and replacement

Using the sampling frame, 10 couples were selected for the weekly LDG sessions with an emphasis on individuals who met the eligibility criteria, lived near the likely site of the LDG group and were willing to commit to weekly participation for 9 months. Replacement of LDG members will occur if a member decides to discontinue participation or if the LDG moderator in consultation with VDRC, EA, and the study PI deem that further participation places the member or another member at risk of violence or other adverse event. Attendance in sessions is monitored weekly to assess “dose” of LDG exposure. Groups with low attendance will receive an in-person supervisory visit from the field monitor and the LDG moderator will follow-up via telephone or in-person with LDG members who are absent to assess barriers to participation and intention to continue.

### FGD recruitment

Family members of LDG members will be recruited for sex-specific FGDs at baseline and again at 9-months post baseline to understand changes in family-based norms on gender equity and IPV, as well as the impact of any project activities and couple-based activism they may have been exposed to. Interested family members will be identified by recommendations made by LDG members. Religious and community leaders will also be recruited into a workshop within which a FGD will be held at the start of the workshop and again 6 months after the conclusion of the workshop to examine changes in their attitudes on gender equity and intimate partner violence, community norms on gender equity and IPV, contextual factors that are occurring in the community, and the community-level impact of the project. Leaders will be identified through recommendations made by stakeholders who are consulted within the study communities.

#### Pair-matching

Each cluster (VDC) was pair-matched based on primary language, caste, percent of literate females per Central Bureau of Statistics (http://www.cbs.gov.np/), and geographic location to minimize contamination. As exact matches are not possible, the final matching procedure was done with VDRC representatives familiar with the local communities best to ensure that the matching process benefited from contextual information not available through public census statistics. Allocation of treatment condition was accomplished by the study Primary Investigator through simple randomization using randomly generated numbers in Excel, with the highest random number per pair being assigned to treatment.

### Allocation concealment mechanisms

Baseline community survey recruitment and administration occurred prior to randomizing study sites in order to minimize bias in recruitment. Further, the informed consent forms did not identify which study-related activities would be implemented in their community. While subsequent survey data collection will inherently come after treatment allocation, the study’s primary outcome stems from the community-based surveys. These individuals will be interviewed by the data collection firm who has no role in the project, enabling the study to keep community survey participants and outcome assessors blind to site allocation. LDG participants cannot be blinded due to the nature of their engagement. Individual recruiters, outcome assessors and data analysts will be blinded to treatment assignment.

### Statistical analysis

The modelling strategy will adhere to intention-to-treat principles. Characteristics of participants and baseline levels of study outcomes across arms will be compared descriptively at baseline to examine potential confounding and to ensure that randomization was successful. Treatment effects will be estimated with generalized logistic mixed models specified to compare differences in primary outcome from baseline to follow-up, and baseline to 18-months post. A random effect for cluster (VDC) will be included to account for within-group clustering, and degrees of freedom will be properly calculated. Time, condition, and time by condition interaction will be fit as fixed effects. To the extent necessary, estimates will be adjusted for socio-demographic characteristics should confounding be detected. Additional quantitative analyses using mixed models will be employed to investigate mediation [39] and adherence. Missing data will be handled using full information maximum likelihood [40].

Transcripts will be generated from audio recordings of FGDs, interviews and open ended IVR responses. An analysis team comprised of the PI and select EA staff will analyse the transcripts using a mixture of deductive and inductive coding approaches, analytic memos, and matrix and network displays [41]. Analysis team members will independently read the transcripts and contribute to a collective draft codebook and series of memos, which are separated into process and analytic components. Analysis will examine the occurrence, dynamics, and pathways to change with a particular emphasis on differences by location, caste, and socioeconomic status. Qualitative analyses will additionally seek to identify any other characteristic or contextual feature that is revealed to be a potential modifying factor of the intervention impact or explanation for null findings. Findings from the qualitative data analysis will inform subsequent quantitative subgroup analyses. Qualitative and quantitative findings will be iteratively fed back into the analysis of the other to generate rich, contextualized results and findings from both processes will be jointly presented in manuscripts to the extent possible.

### Performance monitoring and auditing

The project has an extensive monitoring plan and an accompanying process evaluation. It also has a Technical Advisor assigned by the What Works Global Program who meets weekly with Equal Access’ Technical Advisor for regular updates that are fed back to the Global Program leadership, along with quarterly reports, all of which support ongoing accountability. An independent audit is not anticipated.

### Ethics and dissemination

The study adheres to international guidelines for the protection of participants and staff involved in research on violence against women [42] and has a data and safety monitoring plan which details procedures and reporting obligations regarding expected risks and planned protections, confidentiality and data security, and adverse events with oversight provided by an Independent Monitor who is unaffiliated with the study. This study will be stopped prior to its completion if it is associated with adverse effects that call into question the safety of the participants enrolled. Institutional Review Board (IRB) approval has been received from the University of Minnesota, where the PI was based when the study was funded, Emory University, where the PI is currently based, George Mason University where the co-investigator is based and the Nepal National Health Research Council (NHRC). Permission was also received from the District Development Committees representing Nawalparasi, Kapilvastu and Chitwan.

Written informed consent will be sought from all participants prior to randomization, except for activities related to LDG participation for which recruitment occurred after treatment allocation. VDRC field officers who are involved in recruiting will read the statement the because of the significant number of illiterate individuals in Nepal, most of whom are women. Once participants have agreed to participate in the study, the participant and the person obtaining the consent will sign the consent document. Illiterate individuals will be asked to place an “X” instead of their signature. VDRC field officers will obtain informed consent during the recruitment visit. Informed consent will be reaffirmed by the enumerator by reading a summary of participant rights just prior to administering the survey.

Research indicates that social norms campaigns may have perverse effects [13] including raising awareness of VAWG to the point where it is perceived as being ubiquitous and, thus, more acceptable. We take several steps to address this including engaging community actors in social norms change, such as addressing injunctive rather than descriptive norms, engaging community stakeholders at all levels and involving diverse genders in the development of programming [13, 43]. Additionally, raising awareness of VAWG and available services may encourage women to seek help [44]. The study entails an extensive safety plan, including partnering with key violence advocate and service-providing organizations to ensure the availability of counselling and immediate response. All study participants will be made aware of other resources for women and couples in their areas.

### Dissemination plan

Lessons learned from the study will be shared widely throughout Nepal and among global audiences. To further social change, EA will conduct community forums to review the activities conducted and the results of the intervention. Key results will be made available in Nepali to community members, Nepali media outlets, the study steering committee, and other identified stakeholders to disseminate key findings/messages (including a seminar and fellowship program for Nepali journalists). Lessons from the impact evaluation will be shared via peer-reviewed articles published in the English language in scholarly journals with authorship following International Committee of Medical Journal Editors’ standards. EA will make the evaluation tools, including the baseline and endline surveys and qualitative data questions available to the international community via the internet. EA and the research team will also share the lessons from the impact via local and international media vehicles, such as press releases, social media, and op-eds.

### Protocol amendments

The study is registered with clinicaltrials.gov (NCT02942433). This registration, and all bodies with regulatory oversight will be notified of protocol changes.

## Discussion

This study seeks to rigorously evaluate a multi-component social norms intervention involving radio, LDGs, and community outreach to reduce IPV. Findings may contribute to the evidence-base for strategies to reduce IPV and to shift gender norms. Despite the numerous strengths of the study design and approach, the limitations of the study must be discussed. As with all RCTs, this study aims to have strong internal validity, as opposed to strong external validity. One specific limitation is that only women who have received support and permission from their husbands to participate in the study will be recruited. While this criterion likely restricts the study from including the most vulnerable women, it is meant to protect women in high-risk situations from exacerbated abuse and protect the research team and other participants from potentially dangerous situations. Another important limitation regarding external validity is that the trial excludes participants with easily detectable physical or cognitive impairments, and growing research has underscored how women with disabilities may be particular to VAWG [45–48]. Thus, the research team is fully aware that those who are restricted from participating may have different profiles than those who are able to join. Another important concern is fidelity of intervention delivery given the number of LDGs and their geographic spread which is bolstered by an extensive monitoring and support plan for LDG facilitators.

The impact of the study may also be difficult to discern due to overlap with programs that have similar content being conducted in the study areas. In order to account for the potential impact of other programming, the study will regularly measure participants’ exposure by polling participants during LDGs and interviews and communicating with key stakeholders in the regions of operation. Intermittent and ongoing disruptions in programmatic activities may possibly occur due to fuel shortages and safety issues pertaining to political tensions. The research team will closely monitor these potential disruptions.

In summary, the *Change Starts at Home (Change)* is a multi-component behaviour change communication and community engagement strategy designed to prevent IPV in Nepal. The results of the trial will be immediately useful for governmental, nongovernmental, and donor funded programs targeting partner violence or social norms that underpin it. Findings of the study will also contribute to global knowledge on the effectiveness of media and community engagement as a primary prevention strategy for IPV.

## Abbreviations

EA: Equal access
FGD: Focus group discussion
GEM: Gender equitable men
HIV: Human immunodeficiency virus
ICC: Intra-class correlation
IPV: Intimate partner violence
IVR: Interactive voice response
LDG: Listening and discussion group
RCT: Randomized controlled trial
SBCC: Social behaviour change communication
SMS: Short message service
VAWG: Violence against women and girls
VDC: Village Development Committees
VDRC: Vijaya Development Resource Center
WHO MCS: World Health Organization Multi-country Study on Women’s Health and Domestic Violence

## Footnotes

1 Vox Pop is short for *Vox Populi* (meaning voice of the people) and is a broadcasting term used to describe a range of opinions expressed by the public in the form of informal comments on the same topic or short answers to the same question.
